# Supplementary material for: Metagenome-based vertical profiling of the Gulf of Mexico highlights its uniqueness and far-reaching effects of freshwater input
Source: Appl Environ Microbiol. 2026 Jan 27;92(2):e02589-25. doi: 10.1128/aem.02589-25 (PMC12915328; doi:10.1128/aem.02589-25)
Supplement: Supplemental figures — Fig. S1 to S5. [file aem.02589-25-s0005.pdf]

SUPPLEMENT: Metagenome-based vertical profiling of the Gulf of Mexico highlights its uniqueness and far-reaching effects of freshwater input.

AUTHORS: Roth E. Conrad[1], Despina Tsementzi[2], Alexandra Meziti[2], Janet K. Hatt[2], Joseph Montoya[1], Konstantinos T. Konstantinidis[2]

[1]Ocean Science & Engineering, School of Biological Sciences, Georgia Institute of Technology, Atlanta, GA, USA.

[2]School of Civil & Environmental Engineering, Georgia Institute of Technology, Atlanta, GA, USA.

Supplemental Files

Supplemental File 1: The samples used in this study and their metadata, including physicochemical measurements and bioinformatic analysis of sequence (e.g., Nonpareil) and gene alpha and beta diversity.

Supplemental File 2: Bracken/Kraken taxonomic profiling of the datasets used in this study.

Supplemental File 3: Lists of genes recovered in the datasets used in this study and functional gene content comparisons among the datasets.

Supplemental File 4: Metagenome-assembled genomes (MAGs) recovered from the metagenomes, and their statistics, including taxonomic identification.

Supplemental Figures

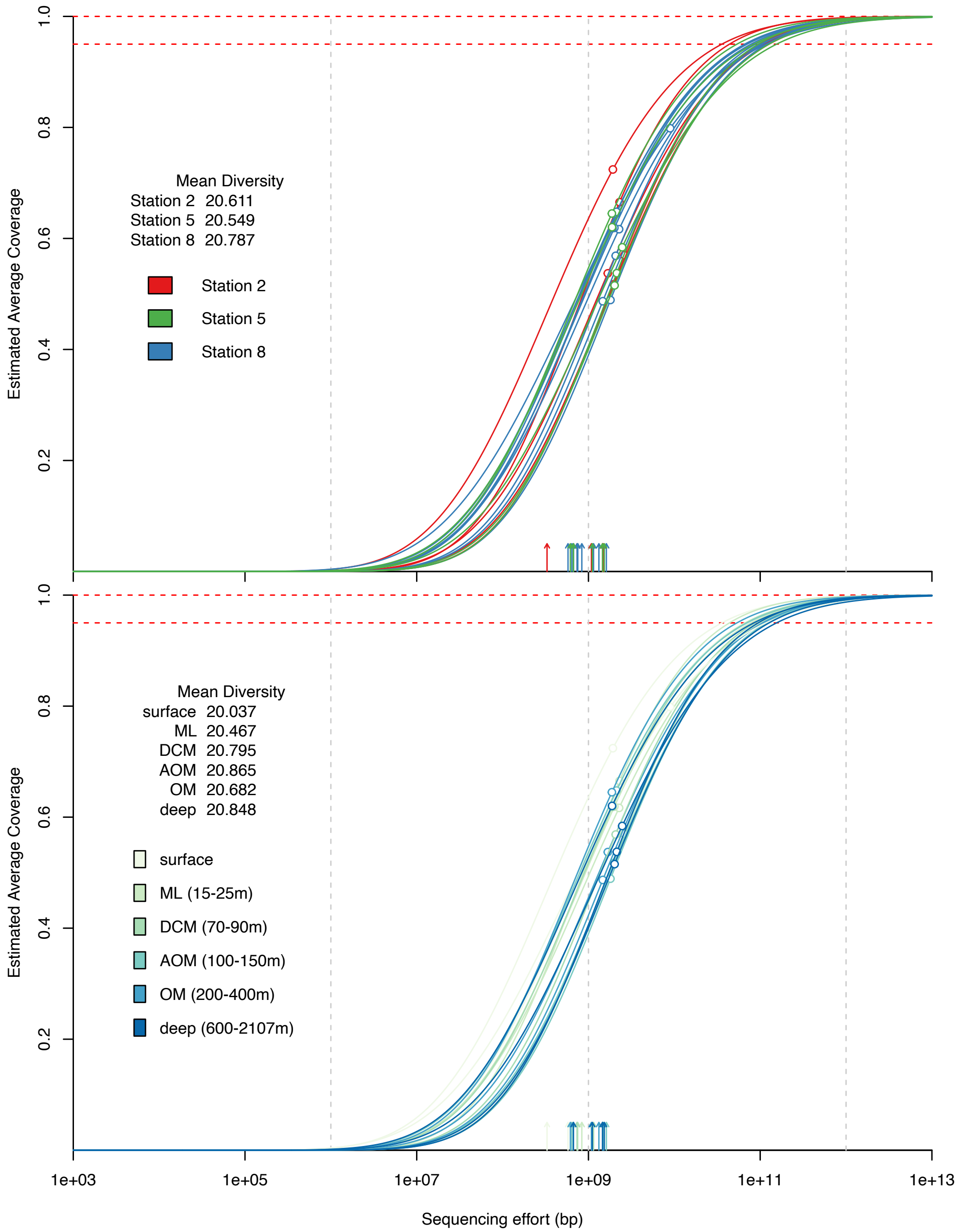

Supplemental Figure 1. Nonpareil curves for GoM metagenomes grouped by sampling station (top) or depth layer (bottom). Nonpareil curves show the average coverage (y-axis) of the estimated sequence diversity of a sample (alpha diversity) versus sequencing effort (x-axis). Each sample has its own curve and the open circles on the curve show the estimated average coverage at the corresponding sequencing effort for that sample. The lower dashed red line marks 95% average coverage of the sequence diversity estimated for a sample and the point where the curve crosses this line indicates the sequence effort estimated to achieve this level of coverage. The arrows at the bottom mark the estimated sequence diversity of each sample.

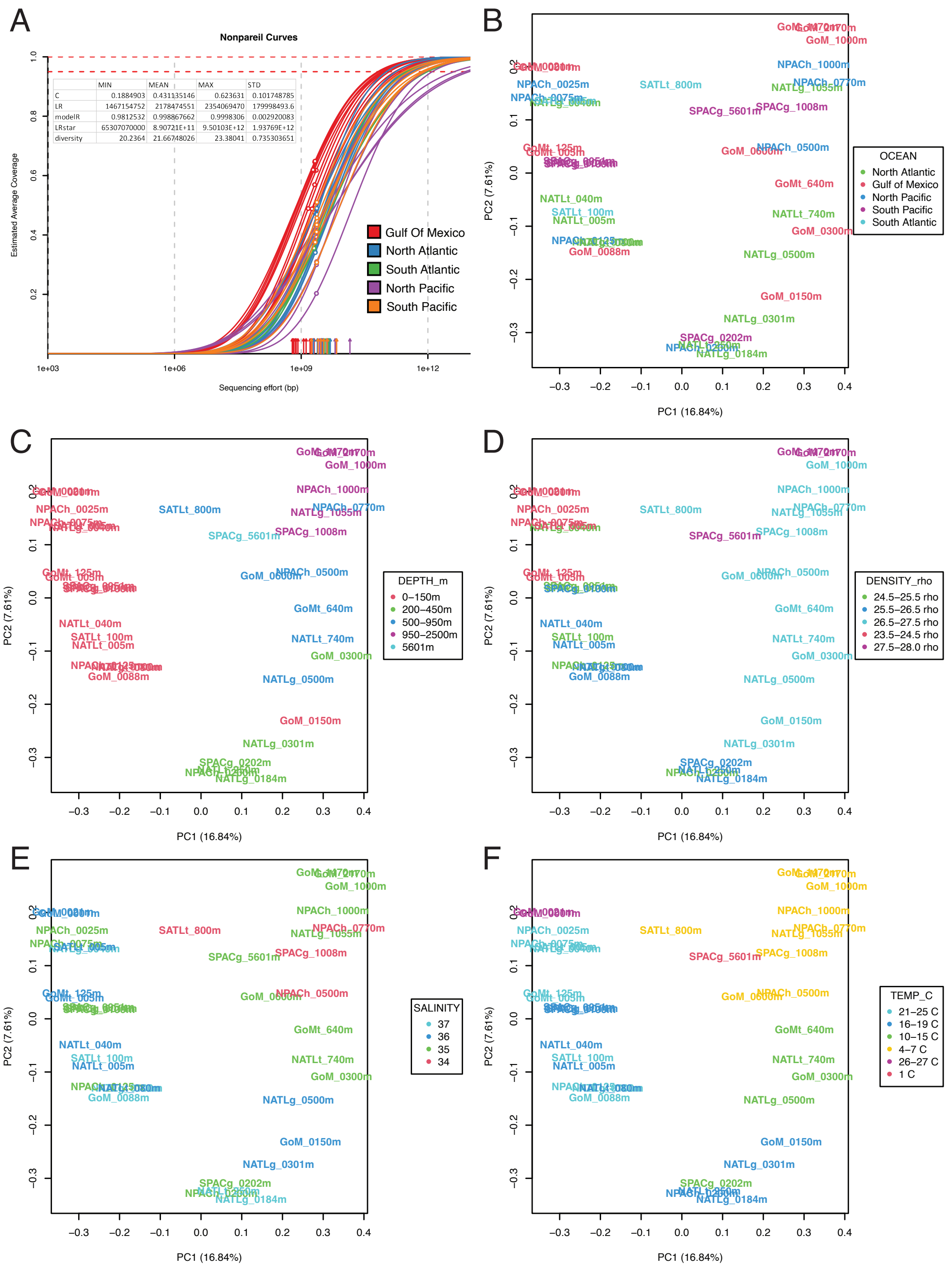

Supplemental Figure 2. Alpha and beta diversity of GoM with other ocean basins. North and South Atlantic and Pacific Ocean samples compared with GoM samples from station 5. Panel A shows Alpha diversity estimated by Nonpareil (x-axis arrows) and Nonpareil curves showing the estimated average coverage (y-axis) based on the sequencing effort (x-axis) for each sample (open circles on curve). Panels B-F show beta diversity estimated with Simka presence/absence Jaccard distance. The same PCoA plot is featured in each sample, but the samples are grouped by Ocean Basin (B), Depth in meters (C), Density in sigma-t (D), Salinity in PSU (E), and Temperature in degrees Celsius (F). The South Atlantic and Pacific Ocean

samples were obtained from NCBI databased (see Supplementary File 1 for accession numbers) and were processed for read quality trimming as described for our GoM datasets in the Materials and Methods section for consistency purposes prior to the Nonpareil and Simka analyses.

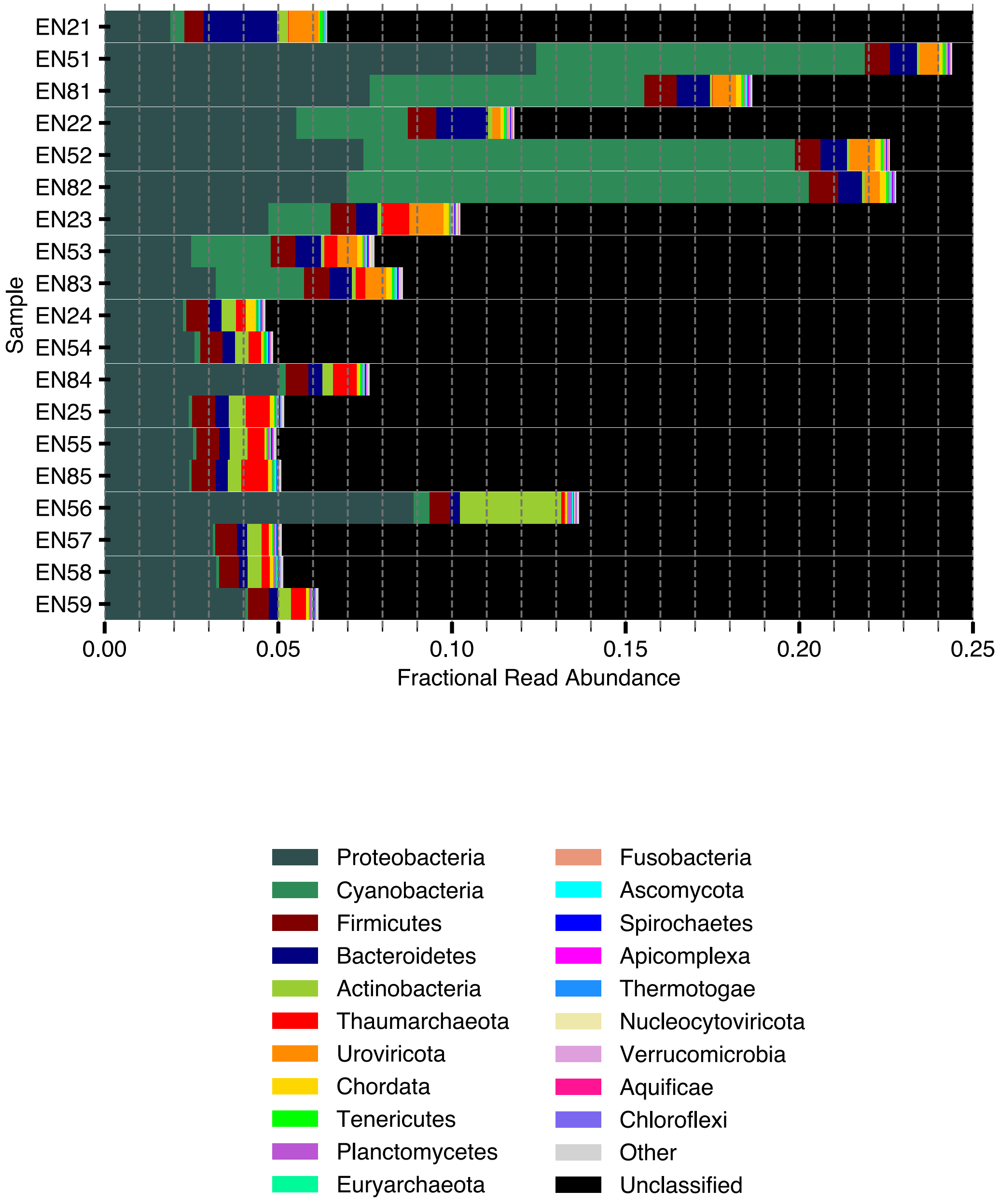

Supplemental Figure 3. Phylum level taxonomic relative abundance. The taxonomic distribution of the top 20 most abundant phylum in each sample as reported by Kraken with Bracken.

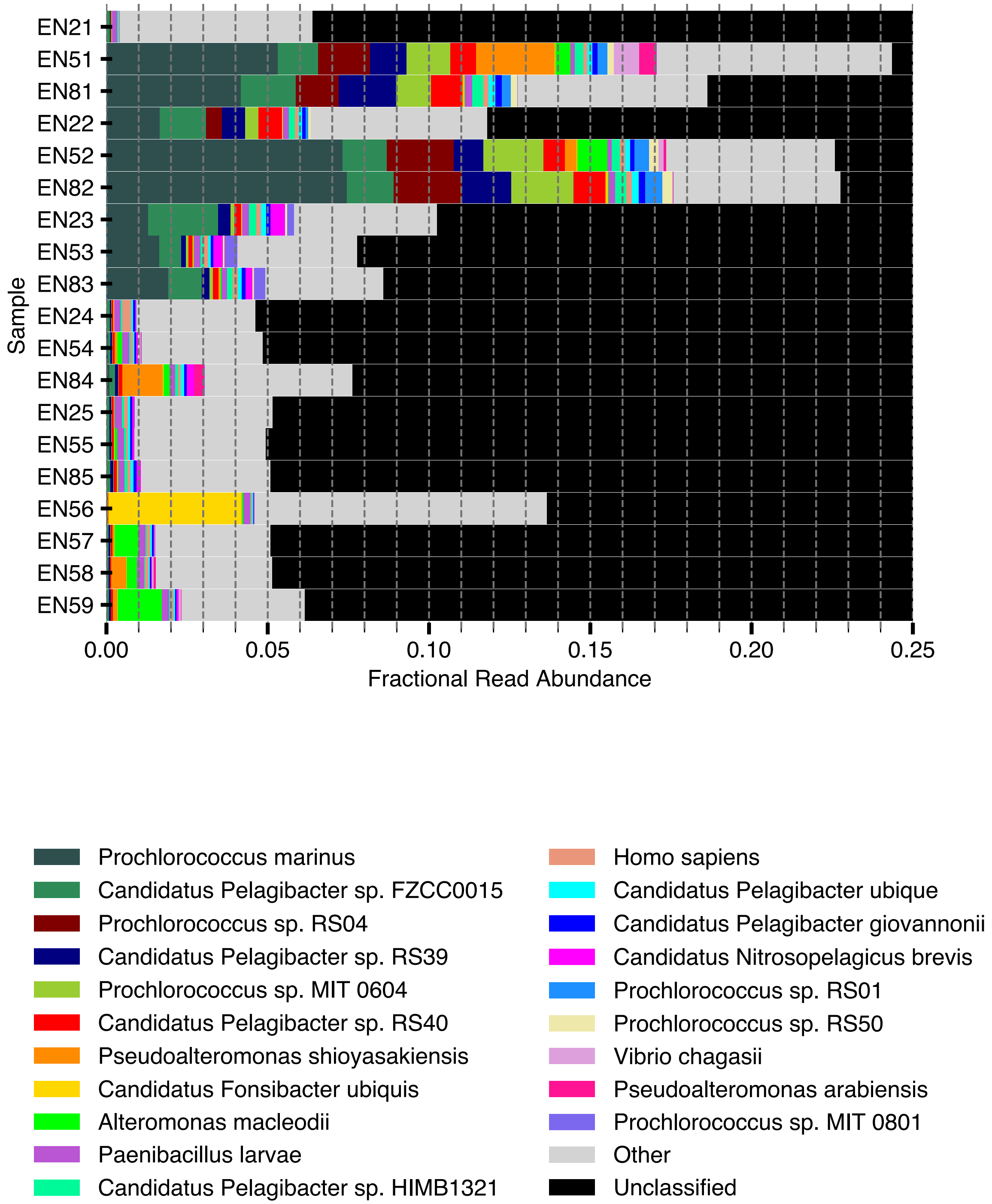

Supplemental Figure 4. Species level taxonomic relative abundance. The taxonomic distribution of the top 20 most abundant species in each sample as reported by Kraken with Bracken.

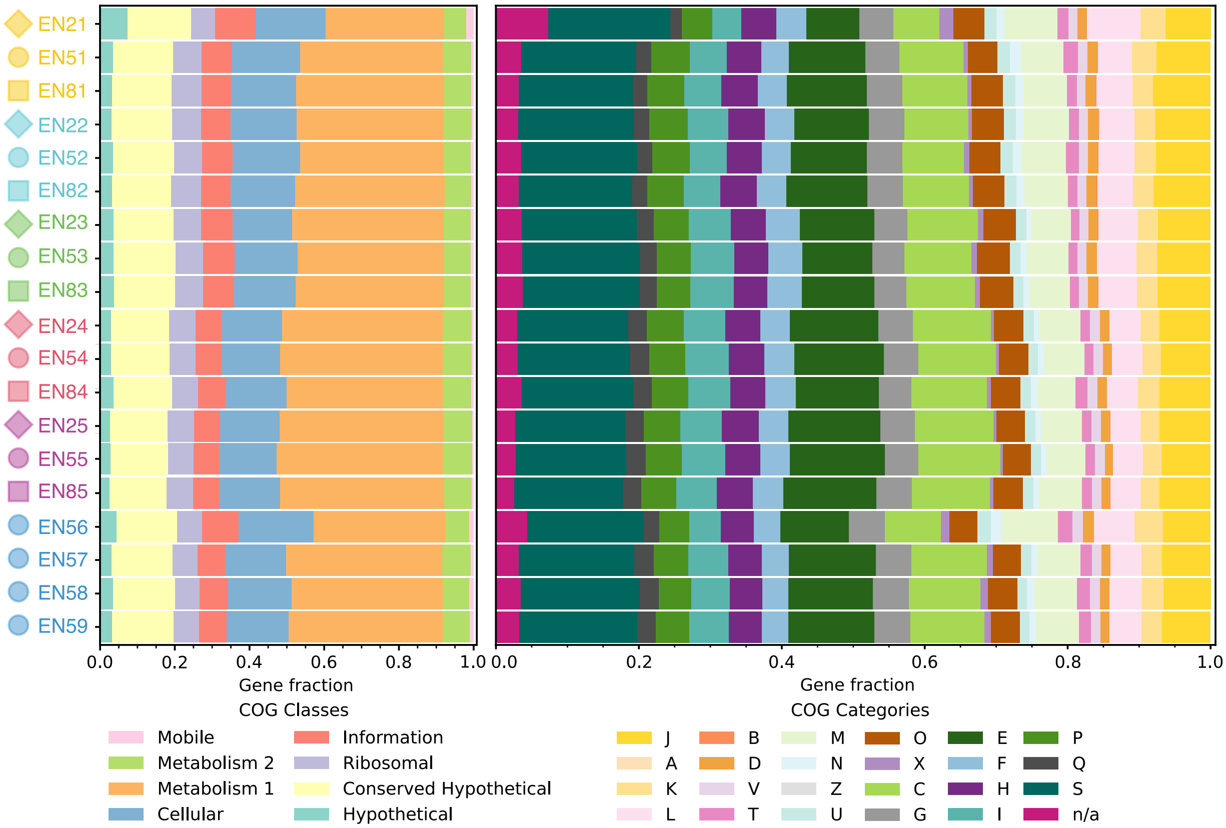

Supplemental Figure 5. Distribution of COG major functional categories in each sample. Predicted CDS from assembled contigs of each metagenome were annotated with EGGNOG mapper. COG Categories (left) and COG Classes (right) were summarized to assess high level functional information. Note: gene fraction includes annotated genes only. 17-38% (mean 22%) of genes predicted for each sample did receive an annotation from EggNog mapper (i.e. did not find a good match in the database).
